# Complete Genome Sequence of the Temperate *Klebsiella pneumoniae* Phage KPP5665-2

**DOI:** 10.1128/genomeA.01118-17

**Published:** 2017-10-26

**Authors:** Gaby Carl, Claudia Jäckel, Josephine Grützke, Stefan Hertwig, Mirjam Grobbel, Burkhard Malorny, Jörg Rau, Annemarie Käsbohrer, Jens A. Hammerl

**Affiliations:** a Department of Biological Safety, German Federal Institute for Risk Assessment (BfR), Berlin, Germany; b Chemical and Veterinary Investigatory Office (CVUA) Stuttgart, Fellbach, Germany; c Institute for Veterinary Public Health, University of Veterinary Medicine, Vienna, Austria

## Abstract

We describe here the genome sequence of the novel temperate *Klebsiella pneumoniae* phage KPP5665-2 isolated from a *Klebsiella pneumoniae* strain recovered from milk in Germany in 2016. The phage exhibited a narrow host range and a siphoviridal morphology. KPP5665-2-related prophage sequences were detected in whole-genome sequencing (WGS) data of various *Klebsiella* species isolates.

## GENOME ANNOUNCEMENT

*Klebsiella pneumoniae* is a Gram-negative species of the family *Enterobacteriaceae*. It is considered an opportunistic pathogen that colonizes a wide range of livestock and wildlife animals but it is also prevalent on plants, in water, and in soil (1). *Klebsiella pneumoniae* mainly infects neonates and elderly or immunocompromised persons (2). The most common diseases it causes are urinary tract infection, pneumonia, and wound or soft tissue infections. *Klebsiella pneumoniae* belongs to the six most significant and dangerous pathogens causing nosocomial infections (*Enterococcus faecium*, *Staphylococcus aureus*, *K. pneumoniae*, *Acinetobacter baumannii*, *Pseudomonas aeruginosa*, and *Enterobacter* species [ESKAPE] pathogens) (2–4). This bacterium was recognized as an important threat to global public health due to its high resistance to antimicrobials (e.g., colistin) (4). Antimicrobial resistance in *K. pneumoniae* is mainly associated with the presence of mobile genetic elements (plasmids) (1). However, until now, the impact of bacteriophages on the transfer of resistance genes is widely unknown in this species. In order to elucidate the role of phages in the evolution of *Klebsiella* spp., the German National Reference Laboratory for Antimicrobial Resistance (NRL-AR) investigates the biology and genetics of temperate phages of *Klebsiella* isolates from food and food-producing animals.

In 2017, phage KPP5665-2 was recovered by mitomycin C (2.5 µg ml^−1^) induction (5) from the *K. pneumoniae* strain CVUAS 5665-2. The strain was collected from a German mastitis milk sample in 2016 by the CVUA-Stuttgart (Germany). Phage particles were concentrated and purified by CsCl density gradient centrifugation (5, 6). The phage exhibited a narrow host range, as plaque formation was observed only on 5 of 66 *K. pneumoniae* strains but not on isolates of other *Klebsiella* species (*n* = 20). Electron micrographs of CsCl-purified phage particles showed that KPP5665-2 possesses an icosahedral head and a long noncontractile tail, typical for the phage family *Siphoviridae*.

Phage DNA was isolated by proteinase K-SDS treatment, as previously described (5, 6). Sequencing of the phage DNA was performed using Illumina MiSeq with 250-bp paired-end reads (5). SPAdes (version 3.5.0) assemblies of trimmed reads resulted in a single contig with a genome coverage of 250-fold per consensus base. Bioinformatic analysis and sequence annotation were performed by using DNA Master (http://phagesdb.org/DNAMaster/). Phage KPP5665-2 exhibited a linear genome of 39,241 bp and a G+C content of 51.6%. A total of 57 protein-coding sequences (CDSs) were predicted, of which 42 are located on the forward strand and 15 on the reverse strand. For 35 protein CDSs, functional predictions could be made. Most products are involved in virion assembly, replication, integration, lifestyle regulation, and host cell lysis. BlastN sequence alignments revealed that the coding region for structural proteins of KPP5665-2 is closely related to cryptic prophage sequences of various *Klebsiella* species, especially *K. pneumoniae*, and of some *Escherichia coli* strains.

Further studies will demonstrate which role this temperate phage can play in the transfer of resistance genes and the lysogenic conversion and fitness of the bacteria. A genomic comparison of a broad range of temperate phages will give deep insights into the genetic diversity of these mobile genetic elements and in their importance in the emergence of new pathotypes.

### Accession number(s).

The complete genome sequence of phage KPP5665-2 was deposited in GenBank under accession number MF695815.
